# Surviving Severe Obstetric Complications: A Population-Based Analysis of Maternal near Miss

**DOI:** 10.3390/medsci14020313

**Published:** 2026-06-12

**Authors:** Ana Paula Jaqueline Crestani, Guilherme Welter Wendt, Caryna Eurich Mazur, Glaucia Osis Gonçalves, Aedra Carla Bufalo Kawassaki, Ana Paula Vieira, Camila Elizandra Rossi, Carolina Panis, Cleide Viviane Buzanello, Dalila Moter Benvegnú, Franciele Ani Caovilla Follador, Geraldo Emílio Vicentini, Gisele Arruda, Léia Carolina Lucio, Lirane Elize Defante Ferreto, Kérley Braga Pereira Bento Casaril, Mariana Abe Vicente Cavagnari, Claudiceia Risso Pascotto

**Affiliations:** 1 Postgraduate Program in Applied Health Sciences, Western Paraná State University (UNIOESTE), Francisco Beltrão 85605-010, Brazil; apjc2101@gmail.com (A.P.J.C.); caryna.mazur@unioeste.br (C.E.M.); aedra.kawassaki@unioeste.br (A.C.B.K.); ana.vieira2@unioeste.br (A.P.V.); carolina.panis@unioeste.br (C.P.); cleide.buzanello@unioeste.br (C.V.B.); dalila.benvegnu@uffs.edu.br (D.M.B.); franciele.follador@unioeste.br (F.A.C.F.); geraldo.vicentini@unioeste.br (G.E.V.); gisele.arruda@unioeste.br (G.A.); leia.lucio@unioeste.br (L.C.L.); lirane.ferreto@unioeste.br (L.E.D.F.); kerley.casaril@unioeste.br (K.B.P.B.C.); mvicente@unicentro.br (M.A.V.C.); 2 Paraná Health State Secretary, Curitiba 80230-140, Brazil; glaucia.osis@sesa.pr.gov.br; 3 Federal University of the Southern Border (UFFS), Realeza 85770-000, Brazil; 4 Department of Nutrition, State University of the Central-West of Paraná (UNICENTRO), Guarapuava 85040-167, Brazil

**Keywords:** epidemiology, maternal health, pregnancy complications

## Abstract

Background: Pregnancies tend to progress without any serious complications. Nonetheless, for a subset of women, obstetric complications may develop, ranging in severity. The most critical of these life-threatening events is referred to as Maternal Near Miss (MNM). To identify the epidemiological and obstetric characteristics, as well as clinical outcomes of MNM cases reported over the year 2021 in the Brazilian state of Paraná. This quantitative, population-based study analyzed 888 notifications that occurred in 2021, obtained from the MNM Notification System. Descriptive statistics and a one-sample Chi-square goodness-of-fit test were applied to the data. Among the women reported, 92.6% were pregnant, the mean age was 29 years, 67.1% identified as white, and 45.2% had preexisting health conditions. Regarding obstetric characteristics, 41.6% were classified as high-risk pregnancies, but nearly one-third (32.3%) of MNM cases occurred in women initially classified as usual risk. The clinical worsening event occurred most frequently during the third trimester (71.9%), and emergency cesarean section was indicated in 60.1% of cases with complete information for this variable. Cesarean delivery predominated over vaginal delivery, with an emergency-to-elective cesarean ratio of approximately 4.7:1. Most women (75%) were discharged after recovery. MNM events are not restricted to women initially classified as high-risk, underscoring the need to strengthen early detection strategies and ensure appropriate management at all levels of care. Improved training of healthcare professionals responsible for reporting and the standardization of MNM monitoring systems in Brazil are also essential.

## 1. Introduction

In general, pregnancies tend to progress without any serious complications, thus resulting in adequate fetal development, a safe delivery, and full maternal recovery by the end of the puerperal period [1]. However, for a subset of women, obstetric complications may develop, ranging widely in severity, a reality which has led the World Health Organization (WHO) to designate the most critical of these events as potentially life-threatening conditions [2]. Although maternal mortality is relatively rare in absolute terms, it remains a critical public health concern, particularly given its largely preventable nature. Yet, for every woman who dies, another 30 develop similarly severe complications and survive [2]. Consequently, for these cases, the WHO defined maternal near miss (MNM) as a woman who nearly died in consequence of serious complications that took place throughout pregnancy, at the time of delivery, or within the 42-day puerperal period [2].

Therefore, identifying women who are experiencing severe complications and potentially life-threatening conditions makes it possible for them to receive adequate and effective treatment, thus preventing maternal death [1]. In this regard, MNM cases provide valuable information on the quality of healthcare and health systems, drawing on medical records and the accounts of surviving women [3,4].

MNM can be considered an indicator of the quality of obstetric services. It serves as a reliable tool for analysis, investigation, and for developing actions across all levels of the health system [1]. According to Brilhante et al. [4], such information is extremely relevant for the training and development of health professionals, largely because timely identification and access to emergency obstetric services are critical for lowering MNM and MM. In Brazil, MNM monitoring is not part of the national notification system. Due to the lack of national standardization, some states use MNM monitoring tools based on WHO criteria [5].

In the state of Paraná, MNM cases are identified by serious circumstances associated with pregnancies, labor, as well as with the puerperium and the subsequent weeks, including cases that result in death. MNM monitoring is used as a source of information to identify weaknesses in the health system and to support policymaking, mobilization, and professional training, aiming to reduce maternal mortality [6]. Monitoring began in 2018, and in 2020, the notification form was revised and incorporated into the Research Electronic Data Capture (REDCap) system [6,7].

Studies indicate that MNM and MM occur due to similar factors, such as lack of access to healthcare services during prenatal care, labor, or the puerperium [1,3]. Other factors include delays or failures by women or their families to recognize the need for professional assistance; poor quality of care; delays in referral for specialized treatment according to severity; and weaknesses or shortages in intensive care units, blood components, blood derivatives, or necessary medications [3,8].

Considering the significant heterogeneity found between nations and among the different regions of Brazil in relation to notification and monitoring of the MNM, this research aims to identify the epidemiological profile, obstetric aspects, and outcomes of MNM cases reported in the state of Paraná, Brazil. Hence, the contributions of this line of research include the importance of identifying risk and possible patterns related to severe maternal morbidity. Also, data gathered from these types of studies can assist in the development of clinical protocol updates, improve obstetric quality, and ultimately provide evidence to help establish a Public Health Policy for MNM events.

## 2. Materials and Methods

This is a quantitative, observational, cross-sectional, population-based, and descriptive investigation. Data were gathered from the official Near Miss Notification System, Department of Health (Secretaria de Estado da Saúde, SESA), Paraná, Brazil. The State is situated in the southern region of the country, covering an area of 199,298.982 km^2^ and comprises 399 municipalities. Its population is estimated to be around 11.8 million inhabitants [9]. The study is described as population-based because it includes all reported MNM cases identified by the state-wide notification system of the Paraná State Department of Health during 2021, rather than a sample drawn from selected facilities. According to SINASC/SIM consolidated data, Paraná registered approximately 141,607 live births in 2021, yielding an MNM ratio of 6.27 per 1000 live births for the cases analyzed in the present study.

After approval by the ethics committee, data from 1078 notifications were extracted from the MNM Notification System, which operates using a form hosted on the REDCap platform [7]. Data were converted into Excel format and provided to the research team by SESA personnel.

**Figure 1:**
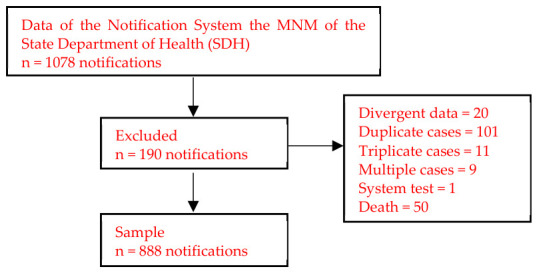
Flowchart depicting sample selection.

All patient identifiers and names were replaced with codes. Data cleaning involved the identification of duplicate entries as well as conflicting information. Thus, instances suggesting double reporting for the same event, the duplicate entry was removed. Cases with conflicting information that could not be resolved through cross-referencing techniques were excluded to ensure data quality. One system test notification was identified and excluded (Figure 1). As such, the current study’s population consisted of all cases of pregnant and puerperal women (up to 42 days postpartum) recorded in the system during 2021. Cases were required to meet the WHO criteria for defining Maternal Near Miss. We excluded repeated cases and also those with divergent data, and records identified as system tests (Figure 1). Notifications corresponding to maternal deaths (*n* = 50) were also excluded from the present analysis, consistent with the WHO operational definition of MNM as a woman who nearly died but survived a complication during pregnancy, childbirth, or within 42 days of termination of pregnancy [2]. The present study therefore characterizes the profile of survivors, which is the conceptual target of MNM surveillance. Because the notification database is not routinely linked to subsequent vital records, women who met MNM criteria at notification but later died cannot be definitively distinguished within the present dataset; this is acknowledged as a limitation. MNM cases were identified by trained healthcare professionals at each notifying facility using the WHO criteria operationalized in the SESA notification form, which encompasses clinical signs and symptoms, laboratory markers of organ dysfunction, and management-based interventions; the full operational definitions used in this study are presented in Supplementary Table S1.

Variables were categorized as follows: (i) sociodemographic characteristics (e.g., maternal age, self-reported race/color, education level, marital status); (ii) Obstetric history (e.g., parity, previous c-sections, number of children); (iii) current obstetric variables (e.g., gestational age, gestational risk stratification, prenatal care coverage, type of delivery and clinical indication); and (iv) clinical outcomes (e.g., ICU admission, length of hospital stay). Gestational risk stratification was determined through the systematic identification of risk factors, grouped into three main categories, namely: (i) usual risk, defined as women without individual or sociodemographic risk factors, no unfavorable reproductive history, and no associated clinical conditions; (ii) intermediate risk, composed of women belonging to vulnerable population groups, such as Black, Brown, or Indigenous; those younger than 15 or older than 40 years; women with low educational attainment (illiterate or with fewer than three years of formal education); and those with a history of fetal or neonatal death in a previous pregnancy; and (iii) high risk, which includes women with preexisting clinical conditions such as chronic hypertension or diabetes mellitus, as well as those who develop obstetric or clinical complications during the current pregnancy, including gestational hypertension, multiple pregnancy, or preterm labor [6]. Moreover, deliveries were evaluated for the use of the particular coding scheme employed by the notification system for the state. Elective cesarean deliveries are referred to as planned deliveries before the onset of labor. Emergency cesarean deliveries indicated the unplanned deliveries necessary for maternal or fetal distress. Intrapartum cesarean deliveries referred to the deliveries that occurred after the onset of labor, for example, failure to progress, yet not indicating the same urgency associated with the “emergency” category. These categories were treated as mutually exclusive to preserve the original classification structure of the notification system [7]. Gestational risk classification (usual, intermediate, high) is assigned prospectively within the Rede Mãe Paranaense protocol at the first prenatal visit and updated whenever new risk factors emerge during prenatal follow-up [6,10]. Thus, the risk stratum recorded in each notification reflects the most recent classification documented in the prenatal record before the MNM event, rather than a retrospective relabeling based on the outcome.

Data were organized in Microsoft Excel and analyzed using IBM SPSS, version 28 (IBM Corp., Armonk, NY, USA). Given the exploratory nature of this study and the novelty of the notification system, the analytical strategy focused on a comprehensive characterization of the MNM cases. Thus, data analysis was as follows: (i) categorical variables (e.g., mode of delivery, outcome of hospitalization, comorbidities, among others) were summarized as absolute frequencies (n) and relative percentages (%); (ii) the continuous variable (maternal age) was reported as mean with standard deviation (SD). Because the present cohort comprises exclusively survivors of severe maternal morbidity, with no comparator group of unaffected pregnancies or maternal deaths, the analytical objective was not to test associations between exposures and disease but to document how marked the imbalance between clinically meaningful categories was within this single survivor cohort. To compare the distribution of clinical characteristics against equal distributions (1:1), one-sample Chi-square (χ^2^) goodness-of-fit tests were performed. Ratios were calculated to describe the relative magnitude of subgroups. The 95% confidence intervals (CIs) for these ratios were derived using the method for the ratio of two binomial proportions. Also, the *p*-value of <0.05 was considered to be statistically significant. These analyses were performed using Jasp, version 0.19.3 (JASP Team, University of Amsterdam, Amsterdam, the Netherlands). A multivariable binary logistic regression was additionally performed to identify factors independently associated with recovery within the MNM cohort. The outcome was operationalized as a binary variable: recovered (i.e., medical discharge after recovery) versus non-recovered at notification (i.e., still hospitalized at the moment of notification, transferred to another facility, or discharged against medical advice); the two cases coded as “Unknown” for outcome of hospitalization were excluded from the regression model. Candidate predictors were entered simultaneously into the model, including demographics (age, comorbidities, parity), maternal age (categorical: ≤18, 19–34, 35+ years; 19–34 as reference), parity (nulliparous, 1–2 previous live children, ≥3; 1–2 as reference), preexisting health conditions (yes vs. no), gestational risk stratification (high vs. usual/intermediate combined), and timing of the MNM aggravation (third trimester vs. all other periods combined). The model was estimated by maximum likelihood; multicollinearity was screened via the generalized variance inflation factor (GVIF ≤ 5 considered acceptable), and overall model fit was assessed using the Hosmer–Lemeshow goodness-of-fit test and the area under the receiver operating characteristic curve (AUC). Cases with missing values on the regression variables were handled by complete-case analysis, with the corresponding sample size for the regression reported transparently in the Results. Adjusted odds ratios (aOR) with 95% CIs and Wald-test *p*-values are reported. The regression analysis and forest-plot visualization were performed in R version 4.4 (R Foundation for Statistical Computing, Vienna, Austria), using the stats, car, ResourceSelection, pROC, and ggplot2 packages.

The study was conducted in accordance with the Declaration of Helsinki and approved by the Ethics Committee in Human Research of the Universidade Estadual do Oeste do Paraná (protocol code 5.600.934, approved on 24 August 2022), and Ethics Committee for studies involving humans of Hospital do Trabalhador/SES/PR (protocol code 5.782.818, approved on 29 November 2022). Patient consent was waived due to secondary data collection.

## 3. Results

A total of 888 cases of MNM were included. The mean age was 29 years (range: 13–48 years). As shown in Table 1, 69.4% were between 19 and 34 years of age. In respect of ethnic and demographic composition, white cases were predominant (67.1%). Moreover, 54.7% of cases reported having between one and eight live children. As for nationality, 96.1% of women were Brazilian, and 3.9% (*n* = 35) were foreign. At the time of hospital admission (entry into care), 92.6% of women were pregnant (*n* = 822), seven were puerperal (0.8%), and in 59 notifications (6.6%), the information was not described on the forms, being considered as unknown (Table 1).

Regarding the gestational risk stratification, 41.6% of the sample was stratified as high risk, 8.4% as intermediate risk, and 32.3% as usual risk. The notifications revealed that 45.2% of women already had some preexisting health conditions. Further, the WHO MNM criteria operationalized for this cohort illustrate that the criteria for the identification of the condition were overwhelmingly influenced by Clinical criteria (90.6%) and Management-based criteria (88.8%). Consequently, the fact that this prevalence has been observed illustrates that the condition, which includes severe pre-eclampsia and hemorrhage, is acutely recognizable through Clinical criteria (e.g., hypertension and shock) that demand an immediate critical intervention (e.g., magnesium sulphate infusion and blood transfusion), while the Laboratory criteria were the least influential at 21.2% for the identification of the condition (as shown in Supplementary Table S1). Cases rarely met a single WHO domain in isolation, but rather showed a high degree of overlap among domains. Thus, for instance, a set of cases associated with hypertensive disorders was also likely associated with both Clinical domains (hypertension) and Management domains (magnesium sulfate). Data analysis showed that there was an average of 2.0 domains per case, the modal value indicated that two domains were most frequently satisfied concurrently, specifically Clinical and Management domains. Additionally, the most severe cases were associated with maximum domain complexity, meeting criteria across all three domains (Clinical, Management, and Laboratory).

Table 2 shows the main preexisting health conditions recorded in the 401 notifications, with Systemic Arterial Hypertension being the most frequently reported, affecting 153 women. The total number of conditions is greater than the number of notifications because 103 women presented with two or more conditions. The “Another” variable (*n* = 150) represents comorbidities with a frequency of less than 1%, including renal, neurological, hepatic, autoimmune, gynecological, and infectious diseases, among others. Allergic conditions (*n* = 34) encompassed self-reported drug, food, and environmental allergies, including a small number of severe atopic conditions (e.g., asthma with documented allergic component); the notification form records the presence of an allergic condition as a binary variable, without specifying the allergen class or severity, so the category should be interpreted as a heterogeneous group. Hematologic disorders (*n* = 24) included anemias of varied etiology (predominantly iron-deficiency and sickle-cell related), thrombocytopenia, and coagulation disorders; the system does not differentiate inherited from acquired conditions. Family history of obstetric, hematologic, or thromboembolic disease is not collected by the SESA notification form and was therefore not available for analysis, which is acknowledged as a limitation of the surveillance instrument. With respect to acute thromboembolic and bleeding episodes during the index event, these correspond to the WHO clinical criteria fields of the notification: coagulation disorders were recorded in 11.0% of cases reporting clinical complications, and shock (which in the SESA dictionary aggregates hypovolemic/hemorrhagic and septic presentations) in 9.6%; no separate field for confirmed thromboembolic events is collected by the system.

In addition to admission status, we also analyzed the timing of aggravation, illustrating that the near-miss event occurred. Although 92.6% of the women were pregnant at the time of hospital admission, the worsening event that led to their MNM classification occurred during the gestational period in 68.01% of cases (*n* = 604), with the third trimester being the most frequent (*n* = 434). Complications during delivery and postpartum were also registered, representing 13.7% and 16.6%, respectively, as shown in Table 3. Out of the cases, 777 reported details on the MNM criteria in the notification form, in which clinical condition was the most prevalent; the most common clinical complication identified in isolation was hypertension (40.77%), followed by dyspnea (14.60%), coagulation disorders (11.02%), shock (9.64%), and heart rate below 40 bpm or over 120 bpm (7.44%).

Even more, 1.7% (*n* = 15) of the notifications were incomplete, resulting in this information being unknown. Regarding the onset and indication of labor, it began spontaneously in 16.3% of valid cases, with 8% requiring induction. Emergency cesarean section was the indication in the majority of cases with complete information for the variable ‘Labor initiation and its indication’ (60.1%). As for the delivery outcome or related interventions, 69.6% were cesarean births and 16.2% were vaginal deliveries.

Out of the 13 abortion cases, 10 were spontaneous with a gestational age under 13 weeks, 2 were induced vaginal deliveries (second trimester), and one was an emergency cesarean section at 36 weeks of gestation. Furthermore, 7.4% of the women remained pregnant after the MNM event. The number of unknown cases (~10% of cases) for Labor initiation and its indication, and for the Outcomes and interventions related to labor, highlights the fragility of the notification system.

Analysis of hospitalization outcomes for MNM cases indicated that 75% of women recovered from the conditions that classified them as MNM and were subsequently discharged from the hospital. At the time of notification, 19.1% remained hospitalized, 4.8% were transferred to another health facility, and seven cases of discharge against medical advice were recorded.

Subsequent analyses were conducted. Hence, a ratio analysis of clinical characteristics pointed to significant disparities in management and outcomes within the MNM sample, aiming to compare the distribution of clinical characteristics against equal distributions (1:1). First, surgical delivery was the predominant mode of birth, with a Cesarean-to-vaginal delivery ratio of 4.3:1 (95% CI: 3.54–5.21; χ^2^ = 270.6, *p* < 0.001). Within the surgical group, the necessity for immediate intervention was noticeable, evidenced by an emergency-to-elective Cesarean ratio of 4.7:1 (95% CI: 3.75–5.83; χ^2^ = 221.5, *p* < 0.001). As for the timing of the adverse event, clinical aggravation was significantly more likely to occur during the antenatal period compared to the postpartum phase (Ratio 4.9:1, 95% CI: 4.12–5.92; χ^2^ = 384.0, *p* < 0.001), stressing the third trimester as the critical window for MNM surveillance. Despite the severity of these cases, the prognosis for recovery was favorable. In other words, for every patient requiring transfer or prolonged hospitalization, approximately three were successfully discharged home (Ratio 3.1:1, 95% CI: 2.68–3.65; χ^2^ = 233.5, *p* < 0.001).

**Figure 2:**
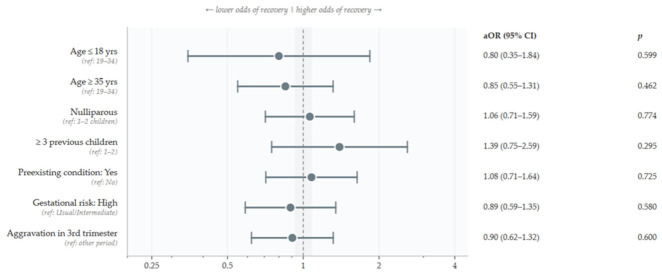
Adjusted odds ratios for recovery within the Maternal Near Miss cohort. Note. All seven predictors were entered simultaneously. Reference categories shown in italics. No predictor reached *p* < 0.05; the AUC near 0.5 indicates that the variables captured by the SESAnotification form do not discriminate recovery trajectories within the MNM cohort, supporting the case for richer surveillance variables. Model fit: *n* = 572 complete cases (events = 416; 72.7% recovered). Hosmer–Lemeshow χ^2^ = 7.74, df = 8, *p* = 0.459; AUC = 0.549 (bootstrap 95% CI: 0.494–0.600); all generalized VIF ≤ 1.27.

To identify factors independently associated with recovery within the MNM cohort, a multivariable binary logistic regression was fitted on 572 cases with complete information on the regression variables; of these, 416 (72.7%) were classified as recovered (medical discharge after recovery) and 156 (27.3%) were classified as not yet recovered at notification (still hospitalized, transferred, or discharged against medical advice). The remaining 316 of the 888 notifications were lost to complete-case selection, primarily because of missing values for gestational risk stratification (*n* = 157, 17.7%), preexisting health conditions (*n* = 118, 13.3%), and parity (*n* = 113, 12.7%). Model adequacy was acceptable: the Hosmer–Lemeshow goodness-of-fit test did not indicate poor calibration (χ^2^ = 7.74, df = 8, *p* = 0.459); all generalized variance inflation factors were ≤1.27, indicating no multicollinearity; and the area under the receiver operating characteristic curve (AUC) was 0.549 (bootstrap 95% CI: 0.494–0.600), which is close to chance discrimination. After mutual adjustment, none of the candidate predictors reached statistical significance at α = 0.05: maternal age ≤ 18 years (aOR = 0.80, 95% CI: 0.35–1.84; *p* = 0.599) and ≥35 years (aOR = 0.85, 95% CI: 0.55–1.31; *p* = 0.462) relative to 19–34 years; nulliparity (aOR = 1.06, 95% CI: 0.71–1.59; *p* = 0.774) and ≥3 previous live children (aOR = 1.39, 95% CI: 0.75–2.59; *p* = 0.295) relative to 1–2 children; preexisting health conditions (aOR = 1.08, 95% CI: 0.71–1.64; *p* = 0.725); high gestational risk relative to usual/intermediate (aOR = 0.89, 95% CI: 0.59–1.35; *p* = 0.580); and clinical aggravation in the third trimester relative to other periods (aOR = 0.90, 95% CI: 0.62–1.32; *p* = 0.600). Adjusted odds ratios with 95% confidence intervals for all predictors are visualized in Figure 2.

## 4. Discussion

The study used notifications recorded in the MNM surveillance system of the state of Paraná, Brazil, in 2021, to characterize the epidemiological profile, obstetric aspects, and outcomes of MNM among pregnant and puerperal women. In general, the results indicate that most epidemiological characteristics observed in this study correspond to the typical profile of the residents covered by health services across the state of Paraná, Brazil. Indeed, in terms of demographics, the majority of patients are white, aged between 19 and 34 years, and multiparous [6]. Although most women were within the typical reproductive age range, the proportion of mothers at age extremes is concerning, as pregnancies in these age groups are linked with a heightened risk for both mortality and MNM [11,12,13,14] and constitute one of the criteria used for gestational risk stratification [15].

Moreover, pregnancies among females younger than 15 or older than 40 years are classified as an intermediate gestational risk factor [6]. According to Diabelková et al. [16], pregnancies in adolescence (defined as the period between 10 and 19 years of age) are more likely to result in preterm births, mainly due to reproductive immaturity, predisposition to infections, social vulnerability, and psychological impacts. Further, scholars noted that women aged 35 years or older can be considered a group at high risk [17], mainly due to a higher prevalence of multiparity and age-related comorbidities.

Fernandes et al. [14] found a high prevalence of MNM among Brown, Black, and Indigenous women living in the North and Northeast regions of Brazil. This difference may be justified by the demographic variety across regions, as the IBGE [18] reports that 76.8% of residents in southern Brazil identify themselves as white.

As for data involving nationality, 3.9% of the women reported in Paraná State’s MNM Notification System were immigrants. According to United Nations data, immigration has increased worldwide in recent years [19]. Although immigrants represented a small portion of the sample, and David et al. [20] found that immigration status did not modify the risk of MNM, it is important to emphasize that immigrant women may face many barriers in accessing healthcare. This is due to unfamiliarity with services, policies, and system infrastructure. Language difficulties, as well as religious and cultural beliefs related to pregnancy and childbirth, may also influence their decision to seek medical care [21]. Studies indicate that delays in seeking care can worsen clinical conditions and increase the risk of MNM and maternal mortality [3,8]. Despite forming a statistically small proportion, the immigrant population could serve as a critical sentinel group for both baseline assessment of the epidemiological profile and follow-up analyses about the inclusivity and responsiveness of the state’s obstetric care network with respect to culturally sensitive protocols.

Further, analysis of the obstetric history showed that multiparous women had between one and eight live children, and the majority had no previous c-section (57.1%). Multiparity has been associated with twice the odds of MNM in primiparous and multiparous women when compared with nulliparous women, possibly due to a progressive increase in obstetric complications and adverse outcomes as the number of pregnancies rises [22]. Additionally, a higher number of previous vaginal deliveries is directly associated with an increased risk of postpartum hemorrhage [15]. Therefore, one of the practical insights from this may include the assessment of multiparous women. These cases should trigger clinical surveillance during the third stage of labor, mostly because these patients already possess vulnerabilities for rapid medical deterioration.

There are three categories of risk factors identified in the classification of gestational risk. Firstly, there is “Usual risk”, which applies to those women without individual or sociodemographic risk factors, unfavorable reproductive history, or associated clinical conditions. Secondly, “Intermediate risk” applies to vulnerable groups, while “High risk” is used for women with preexisting conditions such as chronic hypertension or diabetes, as well as complications during the current pregnancy (e.g., gestational hypertension, multiple or preterm pregnancies) [6]. In fact, the predominance of high-risk pregnant women in the sample was expected, given that MNM events were the focus of this study. However, the finding that 32.3% of the participants were initially classified as low-risk is noteworthy. This demonstrates that severe outcomes can also occur in pregnancies considered to be at low clinical risk. Consequently, results highlight the need for continuous surveillance and high-quality prenatal care for all pregnant women, regardless of initial risk categorization.

The absence of gestational risk stratification in 17.7% of the cases warrants attention, as this process is essential for guiding timely and effective interventions to reduce maternal and infant morbidity and mortality. Failure to classify risk, or inadequate execution of this process, may impede the identification of vulnerabilities and contribute to adverse events, many of which are potentially preventable [23]. Additionally, the right of pregnant women to be informed about the risks they face must be emphasized. Healthcare providers have a professional responsibility to perform accurate risk assessment and maintain complete documentation of gestational information, supporting evidence-based and high-quality obstetric care [10].

Studies show that preexisting comorbidities, especially hypertensive disorders, diabetes, and other chronic diseases, substantially increase the risk of severe maternal morbidity and MNM [24,25,26,27]. This reinforces the importance of rigorous gestational risk stratification, with particular attention to women afflicted with these conditions. Early identification during prenatal care enables better monitoring, optimized obstetric management, and prevention of complications during pregnancy, childbirth, and postpartum. Among the complete MNM notifications analyzed, 68.01% of life-threatening events occurred during pregnancy, mostly in the third trimester (71.86%). Although the presence of comorbidities suggests failures during pregnancy, the available information does not allow precise identification of whether these failures stemmed from delays in seeking care or occurred due to deficiencies in the quality of care provided by services. Many of these outcomes, however, are likely to be preventable.

The results also suggest that, in some cases, labor began vaginally but did not progress as expected, leading to cesarean delivery. Because the present study is descriptive and cross-sectional, the high proportion of cesarean sections observed in this cohort should be read as a description of clinical management decisions made in the context of severe maternal complications, not as evidence that cesarean delivery itself caused, prevented, or worsened those complications; given that all participants by definition met MNM criteria, the design cannot disentangle indication bias (i.e., women whose clinical condition required surgical delivery) from any independent effect of the procedure. A total of 567 cesarean sections were performed, including cases that initially began as vaginal births. Emergency cesarean delivery accounted for 60.1% of these procedures. In this respect, some studies report that cesarean delivery is a lifesaving intervention for the mother, fetus, or both in life-threatening situations [1,28,29,30]. In contrast, Silveira et al. [26], and Kaskun and Greene [31] reported higher rates of admission to Intensive Care Units, sepsis, infections, hemorrhage, and other intra- and postpartum complications (such as curettage, hysterectomy, and laparostomy associated with cesarean delivery or previous surgical births). These findings suggest that, while cesarean delivery may be crucial for preventing maternal death, it may also increase maternal and neonatal morbidity, particularly when performed in high-risk contexts or without strict clinical indication. As such, the dual nature of cesarean deliveries (i.e., protective and harmful) might complicate the manner in which obstetric care is provided throughout health systems marked by significant organizational and regional disparities [28,29]. Additionally, services that lack adequate resources, staffing, and surgical capacity may experience higher rates of complications associated with cesarean deliveries, regardless of whether this type of delivery is clinically indicated. The increase of investment in maternity care delivery models, establishing reliable emergency obstetric care networks, and providing equal access to highly skilled obstetric teams could serve to decrease disparities in maternal outcomes for women who develop life-threatening complications during pregnancy [3,24].

Additionally, given the long-term impacts of cesarean deliveries, such as an increased risk of developing conditions associated with future pregnancies, careful consideration is recommended. Women who experienced maternal near misses and were treated with a cesarean delivery may also be at increased risk for complications associated with future pregnancies. As such, mothers with MNM status and caesarean delivery history should be the focus of postpartum follow-up actions, including comprehensive reproductive planning and health monitoring [3,24].

Regarding hospitalization outcomes, 75% of the women were discharged, 19.1% remained hospitalized, and 4.8% were transferred to other hospital units. Although discharge indicates recovery from a life-threatening complication, it does not necessarily guarantee full clinical resolution. Indeed, survivors may experience physical and psychological sequelae and face higher risks in future pregnancies. Monitoring length of hospitalization, clinical stability at discharge, and post-discharge follow-up is essential to ensure the quality of obstetric care and proper monitoring of the accuracy of MNM data. Undeniably, incorporating structured, evidence-based follow-up protocols into a myriad of maternal health services can enable the early detection of both persistent or emerging conditions, ultimately aiming at improving both maternal wellbeing and safety [31].

Incomplete or inconsistent notifications, including missing data fields and duplicate records, highlight operational weaknesses, as well as possible gaps in professionals’ preparedness to report MNM events. These inconsistencies limit MNM monitoring and represent a significant challenge for research [32]. As epidemiology relies on accurate, consistent, and complete data to identify patterns, measure risks, and guide effective interventions, data quality cannot be compromised. This occurs since the ability to generate reliable epidemiological evidence is weakened, ultimately hindering efforts to improve maternal health outcomes. Consequently, policymakers must define clear roles with respect to MNM reporting.

Another limitation of the study is the absence of key infrastructural and sociodemographic data (i.e., formal education, marital status, occupation, and income) in the official notification forms. These variables are essential for characterizing the epidemiological profile of the affected population and should be incorporated in future revisions of the notification form. As such, the establishment of a standardized national system for MNM notification and monitoring is recommended and urgent, along with the inclusion of MNM in the national list of compulsory notification diseases, injuries, and public health events.

Although most of the files had complete core variable data, there were missing values for some variables, including ‘Number of children’ (*n* = 113, 12.7%) and ‘Pre-existing health conditions’ (*n* = 118, 13.3%). In these cases, missing values were arbitrarily coded as ‘Unknown’ and kept in the dataset. Relating to the outcome measures, the final hospitalization status for the cases was small (*n* = 2; 0.2%), and proportions were computed based on valid cases. Additionally, at the point of notification, a total of 170 women (19.1%) were still hospitalized. Being as the study is a cross-sectional observation of the notification date, the final status of the ongoing hospitalizations couldn’t be confirmed for this study (e.g., specifically whether cases of maternal mortality occurred post-cutoff date for this group of cases who clearly qualified as Maternal Near Miss cases at the time of this analysis). Hence, there is the possibility of a faulty status of the final outcome of survival status for this subset of the study group. Further, the current lack of consistency in terminology, mandatory questions, and diagnostic criteria compromises data comparability and hinders the development of effective public health policies at the national level. Consequently, further research is needed, aimed at detecting gaps in surveillance and reporting systems for MNM events and the overall performance of the healthcare delivery system. The results of these studies can provide stakeholders with knowledge of the differences in comparisons between vulnerable populations and assist in designing targeted interventions for adequate allocation of resources. Data collected via MNM studies could likewise provide evidence-based reasons to enhance monitoring actions of maternal health and inform avenues to improve the methodology of reporting the necessary data.

The multivariable logistic regression brings complementary findings to the descriptive analyses. First, none of the demographic and clinical variables captured by the SESA notification form (maternal age, parity, preexisting conditions, gestational risk stratification, and timing of clinical aggravation) was independently associated with recovery within the MNM cohort, and the model’s discrimination of recovered versus not-yet-recovered cases was close to chance (AUC = 0.549, bootstrap 95% CI: 0.494–0.600). Importantly, it seems that this is not a statistical artifact of low power because with 572 complete cases and 416 events the analysis has substantial power to detect moderate-to-large effects, and all confidence intervals on the adjusted odds ratios are centered close to the null. The most parsimonious reading is therefore not that age, parity, or preexisting conditions are irrelevant to maternal outcomes, but that the variables encoded in the current notification form do not have enough granularity, by themselves, to discriminate the trajectories of women who have already met MNM criteria. Second, this null finding reinforces and quantifies one of the central arguments of the present manuscript: the SESA instrument captures the fact and timing of severe maternal morbidity well, but does not capture the broader set of socioeconomic, organizational, and quality-of-care variables that the literature has shown to drive variation in recovery from severe maternal morbidity. The regression model therefore functions less as a hypothesis test about individual-level risk factors and more as direct empirical evidence for the recommendation, made in the introduction and now substantiated in the Conclusions, that future revisions of the MNM notification form should incorporate education, occupation, marital status, income, food security, mental health history, intimate partner violence, and indicators of timeliness and quality of obstetric care if the surveillance system is to support the analytical goals envisaged for it.

The main strength of this study is its population-based design, covering all MNM cases notified across the state of Paraná in 2021 via a single, structured, REDCap-hosted instrument operated by the State Department of Health. The use of standardized WHO criteria for MNM identification, applied consistently across all notifying services, supports comparability with the broader MNM literature, and the linkage with state-level SINASC denominators allows expression of findings as a population MNM ratio rather than as raw counts. Several limitations should be acknowledged. First, the study is cross-sectional and descriptive, so the associations described should not be interpreted causally; in particular, the high prevalence of cesarean delivery reflects clinical decisions in the face of severe complications and cannot be separated from indication bias. Second, the analyses are restricted to survivors by design; women who died after meeting MNM criteria but before vital-record linkage could be performed cannot be identified within the current dataset. Third, the notification form does not capture several socioeconomic and psychosocial determinants well established as drivers of severe maternal morbidity in the literature, including formal education, occupation, marital status, household income, food security, mental health history, and intimate partner violence. The absence of these variables, together with non-trivial proportions of missing data on existing fields (e.g., gestational risk stratification missing in 17.7% of records), constrains the depth of the epidemiological profile that can be drawn and limits the explanatory power of even the multivariable analysis introduced in this revision. Fourth, the “Allergic conditions” and “Another” comorbidity categories used by the surveillance form aggregate heterogeneous conditions and family history is not captured, which restricts the resolution at which preexisting risk can be characterized. Fifth, women who were still hospitalized at the time of notification (*n* = 170; 19.1%) were retained in descriptive analyses but classified as “not yet recovered” in the regression outcome; this is a conservative coding that may attenuate effect sizes if a substantial fraction of these women later recovered. Finally, the analytical strategy used one-sample chi-square goodness-of-fit tests against equal distributions, which we apply here as descriptive summaries of category imbalance within a single survivor cohort and not as tests of generalizable association; readers should interpret the corresponding *p*-values in that limited sense.

## 5. Conclusions

Among the women notified as MNM, a great majority were pregnant, young, identified as white, and almost half had preexisting health conditions. Regarding obstetric characteristics, the majority were classified as high-risk pregnancies, with the worsening event occurring most frequently during the third trimester, and with most cases indicated for cesarean section. Although high-risk pregnancies represented the largest single category of MNM cases, a substantial proportion—nearly one-third of the total sample—occurred in women initially classified as being at usual risk. These results indicate that MNM can affect a wide range of the obstetric population, not just women with previous risk conditions.

Further, in regard to data quality, important incompleteness was found in the records analyzed, which compromises the ability to fully describe the epidemiological profile of this population. Consequently, there is a need for better practices regarding data collection and potentially adding socioeconomic variables to reporting forms. In addition, improving the completeness of MNM data is crucial for the correct risk stratification and development of sound evidence to inform future health policy planning.

## Figures and Tables

**Table 1:** Characteristics of the Near Miss cases, Paraná, Brazil, 2021.

Variable	*n*	%
Pregnant and puerperal women age (±SD *) years	28.90 ± 6.92 *	
Age group (*n* = 888)		
≤15 years	18	2
16–18 years	36	4.1
19–34 years	616	69.4
35–44 years	214	24.1
>45 years	3	0.3
Unknown	1	0.1
Brazilian Institute of Geography and Statistics Ethnic group (*n* = 888)		
White	596	67.1
Brown or pardo	176	19.8
Black	48	5.4
Asian or yellow	7	0.8
Indigenous	6	0.7
Unknown	55	6.2
Number of children (*n* = 888)		
0	289	32.6
1	216	24.3
2	161	18.1
3	61	6.9
4	33	3.7
≥5	15	1.7
Unknown	113	12.7
Number of previous c-sections (*n* = 888)		
0	507	57.1
1	200	22.5
2	89	10
3	30	3.4
4	3	0.3
Unknown	59	6.7
Brazilian nationality (*n* = 888)		
Yes	853	96.1
No	35	3.9
Obstetric status at the hospital admission (*n* = 888)		
Pregnant	822	92.6
Puerperal	7	0.8
Unknown	59	6.6
Gestational risk stratification (*n* = 888)		
High risk	369	41.6
Intermediate risk	75	8.4
Usual risk	287	32.3
Unknown	157	17.7
Preexisting health conditions (*n* = 888)		
Yes	401	45.2
No	369	41.5
Unknown	118	13.3

* SD = Standard Deviation.

**Table 2:** Preexisting health conditions from Maternal Near Miss cases, Paraná, Brazil, 2021.

Preexisting Health Conditions	*n* = 547 *	%
Hypertension (Systemic Arterial Hypertension)	153	28
Thyroid Disorders	50	9.1
Diabetes Mellitus (Type 1 or 2)	45	8.2
Respiratory Diseases	25	4.6
Allergic Conditions	34	6.2
Hematologic Disorders	24	4.4
Substance Use Disorders	20	3.7
Vulnerable Conditions/Social Vulnerabilities	18	3.3
Cardiovascular Diseases	15	2.7
Mental Health Disorders	13	2.4
Another	150	27.4

Note. * The sample size is greater than 401 because some patients have two or more comorbidities.

**Table 3:** Moment of aggravation and outcomes reported in 2021.

Variables	*n*	%
Period of aggravation of the MNM (*n* = 888)		
In the pregnancy (*n* = 604; 68.01%)		
1st trimester (between weeks 1 to 13)	60	9.92
2nd trimester (between weeks 14 to 27)	110	18.22
3rd trimester (equal or after week 28)	434	71.86
During delivery (*n* = 122; 13.7%)		
Postpartum (*n* = 147; 16.6%)		
Immediate puerperium (From day 1 to day 10 postpartum)	125	14.1
Late puerperium (From day 11 to day 42 postpartum)	20	2.3
After puerperium (After 43 day postpartum)	2	0.2
Unknown (*n* = 15; 1.7%)		
Labor initiation and its indication (*n* = 724)		
Non-induced labor or spontaneous	118	16.3
Induced labor	58	8
Emergency c-section	435	60.1
Elective c-section	93	12.8
Intrapartum c-section	13	1.8
C-section by woman’s request	7	1
Outcomes and interventions related to labor (*n* = 815)		
Cesarean birth	567	69.6
Vaginal delivery	132	16.2
Patients are still pregnant	66	8.1
Hysterectomy	23	2.8
Abortion	13	1.6
Curettage	8	1
The delivery or abortion occurred before arrival at the hospital	6	0.7
Outcome of hospitalization (*n* = 888)		
Doctor’s discharge after recovering	666	75
Hospitalized in the moment of notification	170	19.1
Transfer to another hospital	43	4.8
Patient was discharged against doctor’s advice	7	0.8
Unknown	2	0.2

## Data Availability

Restrictions apply to the availability of these data. Data were obtained from the MNM notification System of the Department of Health of Paraná State, Brazil, and are unavailable due to privacy.

## Supplementary Materials

The following supporting information can be downloaded at: https://www.mdpi.com/article/10.3390/medsci14020313/s1, Table S1: Operationalization of WHO Maternal Near Miss criteria in the Paraná State Department of Health (SESA) notification form, 2021.

## Abbreviations

The following abbreviations are used in this manuscript:

WHO	World Health Organization
REDCAP	Research Electronic Data Capture
MM	Maternal mortality
MNM	Maternal Near Miss
SDH	State Department of Health
SESA	Secretaria Estadual de Saúde
SPSS	Statistical Package for the Social Sciences
IBGE	Instituto Brasileiro de Geografia e Estatística
