# Supplementary material for: Surviving Severe Obstetric Complications: A Population-Based Analysis of Maternal near Miss
Source: Med Sci (Basel). 2026 Jun 12;14(2):313. doi: 10.3390/medsci14020313 (PMC13304291; doi:10.3390/medsci14020313)
Supplement: Supplementary file 1 [file medsci-14-00313-s001.zip › medsci-4275329-supplementary.pdf]

Supplementary Table 1. Operationalization of WHO Maternal Near Miss criteria in the Paraná State Department of Health (SESA) notification form, 2021.

WHO domain	Criterion (WHO 2011)	Operationalization in SESA notification form (2021)
Clinical	Acute cyanosis	Recorded as clinical sign during the index event
	Gasping	Recorded as clinical sign during the index event
	Respiratory rate > 40 or < 6/min	Recorded numerically; flagged if outside reference range
	Shock	Recorded as composite field aggregating hypovolemic/hemorrhagic and septic presentations; no separate field by etiology
	Oliguria non-responsive to fluids/diuretics	Recorded as clinical sign during the index event
	Clotting failure / coagulation disorder	Recorded as clinical sign during the index event
	Loss of consciousness lasting ≥ 12 h	Recorded as clinical sign during the index event
	Loss of consciousness AND absence of pulse/heartbeat	Recorded as clinical sign during the index event
	Stroke	Recorded as clinical sign during the index event
	Uncontrollable fit / status epilepticus	Recorded as clinical sign during the index event
	Jaundice in the presence of pre-eclampsia	Recorded as clinical sign during the index event
Laboratory	Oxygen saturation < 90% for ≥ 60 min	Captured when documented in medical record (variable completeness)
	$PaO_2/FiO_2 < 200$ mmHg	Captured when documented in medical record
	Creatinine ≥ 300 µmol/L or ≥ 3.5 mg/dL	Captured when documented in medical record
	Bilirubin > 100 µmol/L or > 6.0 mg/dL	Captured when documented in medical record
	pH < 7.1	Captured when documented in medical record
	Lactate > 5 mmol/L	Captured when documented in medical record

WHO domain	Criterion (WHO 2011)	Operationalization in SESA notification form (2021)
	Acute thrombocytopenia (< 50,000 platelets/mL)	Captured when documented in medical record
	Loss of consciousness AND presence of glucose and ketoacids in urine	Captured when documented in medical record
Management-based	Use of continuous vasoactive drugs	Recorded as management intervention during the index event
	Hysterectomy following infection or hemorrhage	Recorded as management intervention during the index event
	Transfusion of ≥ 5 units of red-cell concentrate	Recorded as management intervention during the index event
	Intubation and ventilation for ≥ 60 min not related to anesthesia	Recorded as management intervention during the index event
	Dialysis for acute renal failure	Recorded as management intervention during the index event
	Cardiopulmonary resuscitation	Recorded as management intervention during the index event
	Magnesium sulphate infusion for eclampsia / pre-eclampsia management	Recorded as management intervention during the index event

Note. WHO criteria are reproduced from the WHO near-miss approach for maternal health (WHO, 2011). The notification form operationalizes each criterion as a structured field in the REDCap instrument administered by SESA. Laboratory criteria are captured only when the underlying laboratory value is documented in the medical record, which contributes to the lower observed prevalence of laboratory-based criteria relative to clinical and management-based criteria. The full instrument and field dictionary are available on request from the Paraná State Department of Health.
